# Effectiveness of selective digestive decolonization therapy using oral gentamicin for eradication of carbapenem-resistant Enterobacteriaceae carriage

**DOI:** 10.1017/ice.2021.492

**Published:** 2022-11

**Authors:** So Yeon Park, Jin Seo Lee, Jihyu Oh, Seo Hu Lee, Jion Jung

**Affiliations:** 1 Division of Infectious Diseases, Kangdong Sacred Heart Hospital, Hallym University School of Medicine, Seoul, South Korea; 2 Department of Medicine, KyungHee University Graduate School, Seoul, South Korea; 3 Clinical Practice Nurse of Infectious Diseases, Kangdong Sacred Heart Hospital, Seoul, South Korea

## Abstract

**Objectives:**

To evaluate the efficacy of selective digestive decolonization (SDD) therapy using oral gentamicin against carbapenem-resistant Enterobacteriaceae (CRE) colonization and to compare the incidence of novel gentamicin resistance between SDD and non-SDD patient groups.

**Design:**

Retrospective cohort study.

**Setting:**

Acute-care referral center hospital in South Korea.

**Methods:**

Adults aged ≥20 years identified as rectal CRE carriers hospitalized between October 2019 and June 2020 were enrolled. Patients with a <30-day follow-up were excluded. Among CRE carriers, those who received 80 mg oral gentamicin sulfate (Shin Poong Pharmaceutical, Seoul, South Korea) 4 times daily comprised the SDD group and those who did not receive SDD therapy comprised the non-SDD group. CRE decolonization was compared between groups within 15 days, and new gentamicin resistance was assessed.

**Results:**

In total, 73 rectal CRE carriers were identified; 11 patients were lost to follow-up within 30 days and were excluded. Oral gentamicin was administered to 20 of 62 patients. We detected no differences in the basic demographic features between groups. The rate of decolonization within 15 days was higher in the SDD group than in the non-SDD group (70.0% vs 23.8%; *P* = .001). The time to decolonization was significantly shorter in the SDD group. We detected no difference in acquisition of new gentamicin resistance between the groups. No serious adverse events due to oral gentamicin SDD therapy were reported.

**Conclusions:**

SDD therapy using oral gentamicin for CRE-colonized patients may be effective for the decolonization of gut CRE and for the prevention of transmission and subsequent CRE infection.

Increasing infection rates of carbapenem-resistant Enterobacteriaceae (CRE) are considered one of the most urgent threats to public health because carbapenems are used frequently to treat multidrug-resistant isolates. The most common pathogen causing CRE infections worldwide is *Klebsiella pneumoniae.* ^1^ Carbapenem resistance has also been increasingly reported in *Escherichia coli*, *K. oxyoca*, and *Enterobacter cloacae*. Colonization rates vary according to the endemic region and change over time; CRE colonization has been reported in 3%–7% of hospitalized patients.^2–4^ Prolonged inpatient stay increases the risk of exposure to and colonization by CRE. Other risk factors include long-term care facility or intensive care unit (ICU) stay, underlying medical conditions, and previous antibiotic use.^5,6^ CRE colonization has been reported as an important risk factor for CRE infection,^7,8^ which potentially leads to a high morbidity and mortality.^9–11^

Several decolonization studies of CRE carriers have been conducted but have produced conflicting results. Machuca et al^12^ reported that all-cause mortality and CRE infection were reduced significantly with selective digestive decolonization (SDD) therapy, specifically aminoglycosides such as neomycin, streptomycin, and gentamicin. In contrast, Lubbert et al^13^ did not find any significant effect with SDD, specifically gentamicin and colistin, on mortality. An increase in secondary resistance to decolonizing agents has also been reported.^12–14^ Prevention and elimination of CRE colonization might be an effective tool for reducing morbidity and mortality associated with these infections. Decolonization attempts of CRE carriers may also aid infection control and prevent spread to other inpatients.

In this study, we assessed the effect of SDD treatment using oral gentamicin on CRE colonization, and we compared the incidence of new gentamicin resistance between CRE-colonized patients with and without SDD treatment.

## Methods

### Study design and patients

This retrospective cohort study was conducted at Kangdong Sacred Heart Hospital, a 680-bed referral center with 3 ICUs in Seoul, South Korea. This retrospective review included patients with CRE colonization medical records and those with detailed reports in a microbiology laboratory database.

In our hospital, the following infection control strategies are implemented for CRE control: (1) High-risk patients (defined as those hospitalized during the previous 3 months in acute care or long-term care facilities) undergo 2 CRE surveillance cultures at 48-h intervals on admission. (2) All patients admitted to the ICU undergo both CRE surveillance culture on admission—this is repeated once weekly. (3) Patients colonized with CRE were subject to strict cohorting. (4) CRE culture is performed for patients sharing the same room with newly diagnosed CRE carriers. (5) Isolated CRE carriers and patients in contact with CRE carriers observe contact precautions. And (6) screening is performed for follow-up of all patients with a history of CRE colonization on every subsequent admission.

This study included patients aged 20 years or older with CRE colonization. Consecutive CRE-colonized patients identified between October 2019 and June 2020 were included. All patients were followed for at least 30 days. Patients were excluded if they were not followed for a minimum of 30 days. We followed the enrolled patients until CRE decolonization was confirmed, if possible. We classified CRE-colonized patients who received gentamicin SDD therapy into the SDD group and the untreated patients into the non-SDD group. SDD therapy comprised 80 mg gentamicin sulfate (Shin Poong Pharmaceutical, Seoul, South Korea) in solution (8 mg/mL); the solution was administered orally 4 times daily, based on previous studies.^12,14^ The SDD therapy duration was 10–16 days. Rectal swab cultures were performed every 3–7 days thereafter.

Our hospital recommends SDD therapy for CRE-colonized patients to achieve CRE eradication. However, the implementation of SDD therapy was at the discretion of the patient’s attending physician.

This study was approved by the Institutional Review Board of Kangdong Sacred Heart Hospital (IRB No 2020-10-005). The need for informed patient consent was waived due to the retrospective nature of the study.

### Study variables and definitions

Clinical data were extracted from the patients’ electronic medical records and the microbiology laboratory database. The following information was collected: age, sex, underlying diseases, cause of admission, CRE strain susceptibility to gentamicin, use of concomitant systemic antibiotherapy while receiving SDD therapy, prior antibiotic therapy, development of gentamicin resistance, patient’s location when CRE colonization was detected (general ward or ICU), and location before hospitalization (nursing home, nursing hospital, or acute care hospital). CRE was defined as Enterobacteriaceae showing intermediate or resistant susceptibility to ertapenem, imipenem, meropenem, or doripenem irrespective of carbapenemase production. CRE colonization was defined as the presence of CRE in a rectal or stool culture. Decolonization of CRE from the digestive track was defined as a minimum of 3 consecutive negative rectal or stool culture results separated by a period of 3–7 days. When 3 consecutive rectal or stool cultures were confirmed to be negative, the date at which the first negative culture was confirmed was defined as the decolonization date. Decolonization day 15 was defined as the decolonization date within 15 days after CRE colonization was confirmed or SDD therapy was initiated. Day 1 was the day when SDD was initiated (in the SDD group) or the day colonization was detected for the first time (in the non-SDD group).

### Microbiology and antimicrobial susceptibilities

Patient rectal swab or stool specimens were cultured. Isolates from the colonies were identified using the automated VITEK 2 system (bioMérieux, Marcy-l’Étoile, France). Antimicrobial susceptibility testing was conducted using the gram-negative identification card in the VITEK 2 system according to the Clinical and Laboratory Standards Institute guidelines. The modified Hodge test was performed on all CRE isolates as described by the Clinical and Laboratory Standards Institute. For the positive isolates test in the modified Hodge test, a carbapenemase confirmatory test was conducted by polymerase chain reaction.

### Statistical analysis

Normally distributed continuous variables are reported as mean ± standard deviation and compared using the Student *t* test. Nonnormally distributed continuous variables are reported as medians with interquartile ranges (IQRs) and compared using the Mann-Whitney *U* test. Categorical variables were reported as percentages and compared using the χ^2^ test or the Fisher exact test, as appropriate. A multivariate analysis was used to identify independent factors of CRE decolonization. Variables with *P* values <.05 in the univariate analyses were candidates for inclusion in the multivariate analysis. Odds ratios (ORs) were calculated with 95% confidence intervals (CIs). Duration of persistent CRE colonization was calculated as the time from the date of new detection of CRE colonization to the date of CRE decolonization, and the curves were obtained using the Kaplan–Meier method. Comparisons of CRE decolonization between groups were conducted using log-rank tests. All reported *P* values were 2-tailed, and *P* < .05 was considered statistically significant. All statistical analyses were performed using SPSS version 26 software (IBM, Armonk, NY).

## Results

### Study population characteristics

During a 9-month period, 73 patients were identified as CRE-colonized patients; 22 patients received SDD therapy and 51 patients did not. Among them, 11 patients were lost to follow-up within 30 days and were excluded from the study. Overall, 20 patients in the SDD group who received 80 mg oral gentamicin 4 times daily and 42 patients in the non-SDD group were eligible for study inclusion. The baseline characteristics of the patients are presented in Table 1. The mean patient age was 76.4 ± 8.50 years in the SDD group and 69.12 ±13.73 years in the non-SDD group (*P* = .013). There were 13 males (65.0%) in the SDD group and 27 males (46.3%) in the non-SDD group (*P* = .956). Also, 25 patients (40.3%) were admitted from nursing hospitals. Among 62 patients, 25 (40.3%) were bedridden. The most common underlying disease was neurologic disease (Table 1). We detected no significant difference in underlying disease, antibiotic therapy before study enrollment, or concomitant systemic antibiotic therapy during the study period between groups (Table 1). The most concomitantly administered antibiotic was carbapenem (70.0% in the SDD group vs 76.2% in the non-SDD group; *P* = .603). One patient in the SDD group and 7 patients in the non-SDD group received colistin; the difference was not significant (*P* = .258). Also, 6 patients in the non-SDD group received tigecycline; no patient in the SDD group did.

**Table 1.** Baseline Characteristics of the Study Population

Variable	SDD group (n = 20), No. (%)^a^	Non-SDD group (n = 42), No. (%)^a^	Total patients (n = 62), No. (%)^a^	*P* Value
Sex, male	13 (65.0)	27 (46.3)	27 (62.8)	.956
Age, (mean ± SD)	76.4 ± 8.50	69.12 ± 13.73	71.47 ± 12.69	.013
Neurologic diseases	14 (70.0)	23 (54.8)	37 (59.7)	.253
Diabetes mellitus	8 (40.0)	15 (35.7)	23 (37.1)	.744
Chronic renal diseases	5 (24.2)	10 (23.8)	15 (24.2)	.919
Cardiovascular diseases	4 (20.0)	9 (21.4)	13 (21.0)	1.000
Solid cancer	1 (5.0)	9 (21.4)	7 (16.3)	.146
Bedridden state	12 (60.0)	13 (31.0)	25 (40.3)	.051
Medical therapy	17 (85.0)	27 (64.3)	44 (71.0)	.093
Surgical therapy	3 (15.0)	16 (38.1)	19 (30.6)	.082
**Admitted from**				
Nursing home	3 (15.0)	4 (9.5)	7 (11.3)	.671
Nursing hospital	11 (55.0)	14 (33.3)	25 (40.3)	.104
Acute care hospital	5 (25.0)	13(31.0)	18 (29.0)	.629
Location when CRE colonization confirmed				.573
General ward	12 (60.0)	22 (52.4)	34 (54.8)	
Intensive care unit	8 (40.0)	20 (47.6)	28 (45.2)	
Antibiotic exposure within the previous month	18 (90.0)	36 (85.7)	54 (87.1)	1.000
Concomitant systemic antibiotic therapy	20 (100)	40 (95.2)	60 (96.8)	1.000
**Colonizing organisms**				
CPE	10 (50.0)	15 (35.7)	25 (40.3)	.284
Non-CP CRE	10 (50.0)	27 (64.3)	37 (59.7)	
*Klebsiella pneumoniae*	17 (85.0)	30 (71.4)	47 (75.8)	.243
*Escherichia coli*	2 (10.0)	9 (21.4)	11 (17.7)	.478
*Citrobacter spp.*	1 (5.0)	2 (4.8)	3 (4.8)	1.000
*Enterobacter spp.*	1 (5.0)	2 (4.8)	3 (4.8)	1.000
*Serratia marcescens*	0 (0.0)	3 (7.1)	3 (4.8)	.545
Gentamicin sensitivity of colonizing CRE organisms	17 (85.0)	24 (57.1)	41 (66.1)	.030

Note. SDD, selective digestive decolonization; SD, standard deviation; IQR, interquartile range; CPE, carbapenemase-producing Enterobacteriaceae; CRE, carbapenem-resistant Enterobacteriaceae.

a Data are expressed as no. (%) unless otherwise specified.

*K. pneumoniae* (75.8%) was the most common isolate in the rectal carriage of CRE (85.0% of the SDD group vs 71.4% of the non-SDD group; *P* = .243). We detected no significant differences in carbapenemase-producing Enterobacteriaceae isolates between the groups (50.0% of the SDD group vs 35.7% of the non-SDD group; *P* = .284). However, the gentamicin sensitivity rate of colonized isolates was higher in the SDD group than in the non-SDD group (85.0% vs 57.1%; *P* = .030)

### Effectiveness of oral gentamicin SDD therapy

**Fig. 1.**
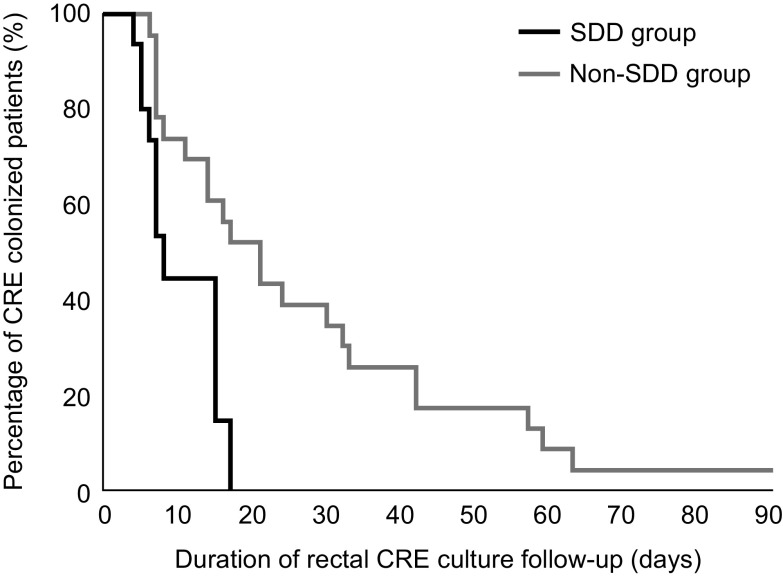
Rectal carbapenem-resistant Enterobacteriaceae (CRE) colonization in the selective digestive decolonization (SDD) group and the non-SDD group. *P* value = .005 by log-rank test.

Patients in the SDD group were treated with oral gentamicin for a median of 14 days (IQR, 12–15 days). The median duration from CRE colonization detection to gentamicin administration in the SDD group was 3 days (IQR, 2.25–4). Decolonization Day 15 and overall duration of decolonization are presented in Table 2. Of the 62 patients included, 24 (38.7%) showed decolonization within 15 days (Table 2). Of the 20 patients in the SDD group and the 42 in the non-SDD group, 14 (70.0%) and 10 (23.8%) had decolonized CRE within 15 days, respectively (OR, 7.467; 95% CI, 2.269–24.572; *P* = .001). In the multivariate analysis, SDD therapy showed a significant association with Decolonization Day 15 (adjusted OR, 30.113; 95% CI, 3.606–524.47; *P* = .002). The median duration of follow-up for all the patients was 36 days (IQR, 30–62). The duration of follow-up in the SDD group was longer than that in the non-SDD group, but this difference was not significant (Table 2). The time to decolonization was shorter in the SDD group than in the non-SDD group (7 vs 19 days; *P* = .001). The Kaplan–Meier curves for persistent CRE colonization according to SDD therapy using oral gentamicin are shown in Figure 1. The SDD group tended to have a higher decolonization rate than the non-SDD group (*P* = .05) (Fig. 1). The CRE decolonization rates were compared 30 days after the end of SDD therapy. Moreover, 55 of 62 patients were followed for >45 days. At 30 days after the end of SDD therapy, the CRE decolonization rate was significantly higher in the SDD group (80% vs 48.6%; *P* = .026). However, there was no significant difference in the CRE decolonization rate between the 2 groups among 47 patients who were followed up for >90 days (94.1% vs 76.775%; *P* = .228).

**Table 2.** Decolonization of Rectal CRE and Acquisition of Gentamicin Resistance According to Oral Gentamicin Use for Selective Digestive Decolonization (SDD) Therapy

Variables	SDD group (n = 20), No. (%)^a^	Non-SDD group (n = 42), No. (%)^a^	Total patients (n = 62), No. (%)^a^	*P* Value
Decolonization within 15 d	14 (70.0)	10 (23.8)	24 (38.7)	.001
Time to decolonization date, median d, IQR)	7.0	19.0	10.5	.001
	(5.5–9.0)	(7.5–37.5)	(7.0–23.25)	
Acquisition of gentamicin resistance	3 (17.6)^b^	4 (16.7)^c^	7 (17.1)^d^	.934
Duration OF Rectal CRE culture follow-up, median d, IQR	36.5 (30–120)	33 (31–70)	36 (30–62)	.786

Note. SD, standard deviation; CRE, carbapenem-resistant Enterobacteriaceae; IQR, interquartile range

a Data are expressed as number (%) unless otherwise specified.

b Only 17 patients with susceptibility to gentamicin were analyzed.

c Only 24 patients with susceptibility to gentamicin were analyzed.

d Only 41 patients with susceptibility to gentamicin were analyzed.

CRE infection occurred in 5 patients (8.1%), with 1 in the SDD group and 4 in the non-SDD group (5.0% vs 9.5%; *P* = 1.000). Death-related CRE infection occurred in 1 patient. The acquisition of gentamicin resistance is shown in Table 2. The gentamicin-resistant isolate rate was 33.9% (21 of 62). The acquisition rate of gentamicin resistance was 11.3% (7 of 41). We detected no significant difference in the newly acquired gentamicin resistance rate during the follow-up between groups (17.6% in the SDD group vs 16.7% in the non-SDD group; *P* = .934). In the SDD group, there were no adverse events, such as nausea, vomiting, diarrhea, or abnormal blood test results.

We compared demographic factors and SDD therapy according to whether CRE decolonization occurred within 15 days (Table 3). Admission from nursing hospitals was more common in nondecolonized patients (Table 3). SDD therapy using oral gentamicin was more prominent in decolonized patients (Table 3). We analyzed SDD therapy and admission from nursing hospitals in a multivariate analysis. SDD therapy was an independent factor associated with decolonization day 15 (58.3% vs 15.8%; adjusted OR, 30.113; 95% CI, 3.606–524.47; *P =* .002), whereas admission from nursing hospitals was an independent risk factor for persistent CRE colonization (Table 3).

**Table 3.** Analysis of Variables and Selective Digestive Decolonization (SDD) Therapy Associated With Decolonization of CRE Within 15 Days

Variable	Decolonized Patients (n = 24), No. (%)^a^	Nondecolonized Patients (n = 38), No. (%)^a^	*P* Value	Adjusted ORs (95% CI)	*P* Value
Sex, male	15 (62.5)	25 (65.8)	.792		
Age, (mean ± SD)	74.42 ± 10.06	69.61 ± 13.91	.956		
Diabetes mellitus	7 (29.2)	16 (42.1)	.304		
Neurologic diseases	12 (50.0)	25 (65.8)	.217		
Chronic renal diseases	4 (16.7)	11 (28.9)	.271		
Cardiovascular diseases	4 (16.7))	9 (23.7)	.509		
Cancer	6 (25.0)	4 (10.5)	.131		
Bedridden state	8 (33.3)	17 (44.7)	.373		
Medical therapy	16 (66.7)	28 (73.7)	.553		
Surgical therapy	9 (37.5)	10 (26.3)	.352		
**Admitted from**					
Nursing home	2 (15.4)	2 (6.7)	.572		
Nursing hospital	2 (15.4)	18 (60.0)	.009	0.065 (0.008–0.551)	.012
Acute-care hospital	3 (23.1)	8 (26.7)	1.000		
Received care in the intensive care unit	12 (50.0)	16 (42.1)	.543		
Antibiotics exposure within the previous month	23 (95.8)	31 (81.6)	.136		
Concomitant systemic antibiotic therapy	23 (95.8)	37 (97.4)	1.000		
Gentamicin resistance	6 (25.0)	14 (36.8)	.331		
Acquisition of gentamicin resistance	0 (0.0)	8 (26.7)	.082		
Gentamicin SDD therapy	14 (58.3)	6 (15.8)	.001	30.113 (3.606–524.471)	0.002
Colonizing organism					
CPE	7 (29.2)	18 (47.4)	.155		
*Klebsiella pneumoniae*	15 (62.5)	32 (84.2)	.052		
*Escherichia coli*	4 (16.7)	7 (18.4)	.860		
*Citrobacter* spp	1 (4.2)	2 (5.3)	1.000		
*Enterobacter* spp	3 (12.5)	0 (0.0)	.054		
*Serratia marcescens*	1 (4.2)	2 (5.3)	1.000		

Note. OR, odds ratio; CI, confidence interval; SD, standard deviation; SDD, selective digestive decolonization; CRE, carbapenem-resistant Enterobacteriaceae; CPE, carbapenemase-producing Enterobacteriaceae.

a Data are expressed as number (%) unless otherwise specified.

## Discussion

Intestinal CRE colonization is the main source of CRE transmission; it is associated with a substantial risk of subsequent CRE infection.^9^ In this study, patients who received SDD therapy using oral gentamicin showed a 70.0% eradication rate, compared to 23.8% in nontreated patients. In addition, the time to decolonization was shorter in the SDD group than in the non-SDD group. Despite the small number of patients analyzed in this study, our results are similar to those of previous studies.^14–16^ Several reports have suggested that decolonization therapy with oral, nonabsorbed antibiotics was effective in eradicating intestinal carriage of CRE and decreased mortality from subsequent CRE infection.^12,16,17^

Nonabsorbing antibiotics for SDD therapy are selected by considering CRE sensitivity to antibiotics and systemic adverse effects.^16,17^ SDD therapy using oral gentamicin was demonstrated to be effective in the gut decontamination of CRE in several studies,^12,15,18,19^ with low serum levels when administered orally.^14^ Because the systemic absorption of oral gentamicin is low, we assumed that there would be fewer systemic effects. The use of oral gentamicin for gut decolonization of CRE has few contraindications. Thus, oral gentamicin therapy may be ideal for this treatment due to its rapid bactericidal activity in vitro against gentamicin-susceptible CRE strains, the virtual absence of systemic activity and toxicity when administrated orally, and its low activity against intestinal flora, especially anaerobes.^16,18^ In this study, there were no adverse events, such as acute renal injury or nausea and vomiting, associated with oral gentamicin therapy.

Notably, gut colonization is the main source of CRE epidemic dissemination. Most patients with CRE infection were colonized before they developed infection by CRE, and the rates of subsequent CRE infection in patients found to be colonized by culture screening was 9% overall and was 27% among patients in ICUs.^17^

One of the problems with SDD therapy using oral gentamicin is the acquisition of new gentamicin resistance. Several studies have demonstrated increasing gentamicin resistance after oral gentamicin therapy.^12,13^ In our study, the acquisition of gentamicin resistance was 23.1% in the SDD group and 26.1% in the non-SDD group. We detected no significant difference in the acquisition of gentamicin resistance between the 2 groups, and the acquisition of gentamicin resistance did not significantly affect decolonization day 15.

Furthermore, SDD therapy showed a significant effect on early decolonization or decolonization within 15 days. However, we detected no significant difference in decolonization between groups during the entire follow-up period. This may have been due to the occurrence of spontaneous CRE decolonization, as has been demonstrated in other studies.^20–22^ Spontaneous decolonization of CRE occurs in 65% of patients after ∼6 months and in 74% within 1 year.^20,21^ With the implementation of SDD therapy, early decolonization of CRE may prevent CRE dissemination, could promptly release patients from quarantine and contact precaution, and may allow early discharge from acute-care hospitals. In CRE-colonized patients, early CRE decolonization may prevent CRE infection, which is difficult to treat.

This study had several limitations. It was performed at a single center and the results were analyzed retrospectively. Some selection bias might have affected CRE colonization or decolonization, despite our best efforts to reduce this. For example, neurologic diseases, bedridden state, and hospitalization at nursing hospitals were closely related to each other, and these factors may have influenced outcomes. Moreover, the decision to perform SDD therapy was at the discretion of the attending physician for each patient, which may have led to selection bias. In the SDD group, there was a delay in SDD therapy initiation after CRE detection (median, 3 days), which may have caused some bias in the results. Second, we used a rectal or stool culture as a standard for decolonization. This may have overestimated decolonization of CRE because of the lower sensitivity of culture methods compared to polymerase chain reaction.^19^ Third, the number of patients in the SDD group was small, which may have influenced the evaluation of the effectiveness of SDD therapy. For these reasons, further prospective analyses with larger populations are needed to confirm the effectiveness of SDD therapy using gentamicin.

In conclusion, SDD therapy with oral gentamicin is safe and potentially effective. Early decolonization might be useful in patients with CRE colonization to shorten isolation procedures and reduce bed occupancy in hospitals with limited resources. In addition, SDD therapy with oral gentamicin is a therapeutic option that can act as a complementary approach for reducing the risk of severe infection with the notoriously difficult-to-treat CRE.
